# Emerging mechanisms of organ crosstalk: The role of oxylipins

**DOI:** 10.1111/nbu.12726

**Published:** 2024-12-10

**Authors:** Helena Lucy Fisk, Saame Raza Shaikh

**Affiliations:** ^1^ Faculty of Medicine, School of Human Development and Health, Southampton General Hospital University of Southampton Southampton UK; ^2^ NIHR Southampton Biomedical Research Centre University Hospital Southampton NHS Foundation Trust and University of Southampton Southampton UK; ^3^ Department of Nutrition, Gillings School of Global Public Health and School of Medicine University of North Carolina at Chapel Hill Chapel Hill North Carolina USA

**Keywords:** Cardiosplenic, immunity, inflammation, lipids, metabolism, Omega‐3

## Abstract

There is growing interest in the role of oxylipins in the pathophysiology of several diseases. This is accompanied by a limited but evolving evidence base describing augmented oxylipin concentrations in a range of complications including cardiovascular disease, obesity, liver disease and neurological disorders. Despite this, literature describing oxylipin profiles in blood and multiple organs is inconsistent and the mechanisms by which these profiles are altered, and the relationships between localised tissue and circulating oxylipins are poorly understood. Inflammation and immune response associated with disease requires communication across organs and physiological systems. For example, inflammation and comorbidities associated with obesity extend beyond the adipose tissue and affect the vascular, hepatobiliary and digestive systems amongst others. Communication between organs and physiological systems is implicated in the progression of disease as well as the maintenance of homeostasis. There is emerging evidence for the role of oxylipins as a mechanism of communication in organ crosstalk but the role of these in orchestrating multiple organ and system responses is poorly understood. Herein, we review evidence to support and describe the role of oxylipins in organ crosstalk via the cardiosplenic and gut‐link axis. In addition, we review emerging mechanisms of oxylipin regulation, the gut microbiome and modification using nutritional intervention. Finally, we describe future perspectives for addressing challenges in measurement and interpretation of oxylipin research with focus on the host genome as a modifier of oxylipin profiles and response to dietary lipid intervention.

## INTRODUCTION

Oxylipins are a large group of lipid signalling molecules produced by oxygenation of monounsaturated fatty acids (MUFAs) and polyunsaturated fatty acids (PUFAs) by enzymatic (cyclooxygenase [COX], lipoxygenase [LOX] and cytochrome P450 [CYP450]) and non‐enzymatic reactions (Dyall et al., 2022; Misheva et al., 2022; Parchem et al., 2024). Oxylipins include prostaglandins (PGs), leukotrienes, thromboxanes and hydroxy‐eicosatetraenoic acids (HETEs) amongst others from the omega‐6 PUFA arachidonic acid (AA). Oxylipins can also be synthesised from long‐chain omega‐3 PUFAs (LC n‐3 PUFAs) which include hydroxy‐eicosapentaenoic acids (HEPEs), hydroxy‐docosahexaenoic acids (HDHAs) and specialised pro‐resolving molecules including resolvins, protectins and maresins. These molecules play a role in immune (innate and adaptive) and inflammatory signalling pathways and as such, have been associated with the pathophysiology of several inflammatory‐linked diseases and have been most notably reported in the context of obesity (Claria et al., 2012; Crouch et al., 2018; Fisk et al., 2021, 2022; Jurado‐Fasoli et al., 2022; Lopez‐Vicario et al., 2019; Neuhofer et al., 2013; Pauls, Du, et al., 2020; Pawelzik et al., 2019) and metabolic syndrome (Arvind et al., 2020; Kalveram et al., 2021; Miao et al., 2021; Misheva et al., 2022; Pawelzik et al., 2019; Tuomisto et al., 2022), pulmonary inflammation and disease (Kilburg‐Basnyat et al., 2018; Lundström et al., 2011; Virk et al., 2022), neurological disorders (Borkowski et al., 2021; Borkowski, Taha, et al., 2021; Dyall et al., 2022; Zhao et al., 2011) and cardiovascular disease (CVD) (Dyall et al., 2022; Huang et al., 2020; Miao et al., 2021; Nayeem, 2018). Oxylipins are most commonly measured in blood and reflect systemic inflammation; however, profiling within organs and physiological systems is gaining clinical interest to advance our understanding of the role of these molecules within the local environments and how this links with systemic inflammation. For example, metabolic syndrome affects several tissues and organs including the adipose tissue, liver, intestines, brain and the cardiovascular system, accompanied by changes to blood markers of health. Despite increased interest in tissue oxylipin profiles and crosstalk between organs and physiological systems, the role of oxylipins in co‐regulation of multiple inflammatory environments is not well understood.

There is increasing clinical interest in targeting these molecules as a route to reduce inflammation and associated negative health outcomes. The most investigated intervention to target oxylipin signalling is the use of long‐chain omega‐3 PUFAs (LC n‐3 PUFAs) (Devassy et al., 2016; Dyall et al., 2022; Fisk et al., 2021, 2022; Gabbs et al., 2021; Ostermann & Schebb, 2017; Rossmeisl et al., 2018; Schmocker et al., 2018). Oxygenation of LC n‐3 PUFAs gives rise to a class of oxylipins termed the specialised pro‐resolving mediators (SPMs) derived from long‐chain PUFAs, notably eicosapentaenoic acid (EPA) and docosahexaenoic acid (DHA) (Dyall et al., 2022; Misheva et al., 2022; Parchem et al., 2024). These LC n‐3 PUFA‐derived SPMs, encompassing the protectins, maresins and resolvin families, have inflammation‐resolving capabilities (Serhan et al., 2008, 2009, 2015). Use of LC n‐3 PUFA has been shown to alter oxylipin profiles in several organs including the liver (Naoe et al., 2019), adipose tissue (Fisk et al., 2021, 2022; Naoe et al., 2019), heart (Naoe et al., 2019), lung (Naoe et al., 2019) and in plasma; however, how alteration of localised oxylipins affects the regulation of physiological system crosstalk (e.g. metabolic and circulatory or digestive and respiratory) is not understood. This is critical to address as many studies continue to not detect or have limited detection of SPMs, which may be driven by a lack of tissue‐specific analysis.

There is emerging evidence for the regulation of immunity and cardiometabolic health via the cardiosplenic axis (Hiraiwa et al., 2022); oxylipin signalling may play a key role in communication within this axis but this is not known. Furthermore, there is emerging evidence for the regulation of oxylipins by the intestinal microbiome (Avila‐Roman et al., 2021; Roussel et al., 2024) which links several physiological systems. Here we will review evidence for the role of oxylipins in inflammatory signalling crosstalk within the cardiosplenic axis and metabolic gut‐linked axis' including brain, adipose and liver. In addition, we will review updated evidence for the use of LC n‐3 PUFAs to modulate oxylipin signalling and the gut microbiome.

## CARDIOSPLENIC AXIS

The cardiosplenic axis refers to the relationship between the spleen and cardiac system, integrating both cardiovascular and immune responses. This relationship was first identified in 1949 when electrical stimulation of splenic nerves restored ventricle function in dogs (Hiraiwa et al., 2022; Rein & Dohrn, 1951). Attention declined until 2009 when it was observed that splenic activation following myocardial infarction increased production of, and mobilised, splenic monocytes which infiltrated the myocardium (Dutta et al., 2012; Hiraiwa et al., 2022; Swirski et al., 2009). The spleen is the second largest lymphoid organ in the body with a plethora of immune cells including neutrophils, monocytes, leukocytes, dendritic cells, macrophages, B and T cells, residing within organ tissues (Lewis et al., 2019). The spleen therefore plays a central role in immune surveillance and response, including the deployment of immune cells to tissue sites and injury, including the heart (Swirski et al., 2009).

Damage to cardiac tissues results in immune cell activation and relocation to the site of injury which is mediated through crosstalk between the spleen and cardiac tissues (Hiraiwa et al., 2022). Splenic metabolic activity and remodelling occur in response to damage, leading to changes in the composition of splenic‐derived immune cells including activated (inflammatory) splenic macrophages which are recruited to the heart (Emami et al., 2015; Hiraiwa et al., 2022; Kercheva et al., 2022). Resultant cardiac remodelling can be adaptive, suppressing cardiac inflammation and supporting normal cardiac rhythm, or lead to cardiac fibrosis and worsening cardiac function (Hiraiwa et al., 2022).

### Mechanisms of communication of the cardiosplenic axis

Changes to immune cell populations and activation can result in altered inflammatory profiles of cells including changes to cytokine signalling, a route of communication between the spleen and heart. Activation of the beta‐adrenoreceptor (sympathetic nervous system) during cardiovascular events promotes splenic leukocyte mobilisation and increased splenic interleukin (IL)‐10 mRNA expression which has been associated with reduced infarct size in mice (Tian et al., 2018). This suggests splenic IL‐10 may have a cardioprotective effect and is a key communicatory signal between the spleen and the cardiovascular system (Markowski et al., 2013; Tian et al., 2018). On the other hand, tumour necrosis factor‐alpha (TNF‐α) and IL‐1β secreted from activated migratory splenic immune cells and those resident in cardiac tissues are implicated in promoting cardiac inflammation and worsening function (Li, Chen, & Wang, 2021; Tschope et al., 2021). What remains less well‐described is the role of other inflammatory cell‐derived signals in cardiosplenic crosstalk such as oxylipins.

Through modulating immune cell behaviour, oxylipins may indirectly influence cardiovascular health through their effects on splenic immune function. Additionally, immune cell‐derived oxylipins themselves can contribute to the local and systemic oxylipin milieu within the cardiosplenic axis. Oxylipins also exert direct effects on vascular function, including regulation of vascular tone, endothelial function and vascular inflammation (Campbell et al., 1996; Goldman et al., 1983; Honda et al., 1999; Nakao et al., 1982; Nayeem, 2018; Nayeem et al., 2009). These effects are mediated through interactions with various receptors and signalling pathways within the cardiovascular system (Nayeem, 2018).

A comprehensive review of the role of oxylipins in CVD by Nayeem et al. (2009) eloquently describes the opposing roles of the oxygenated AA metabolites, epoxy‐eicosatrienoic acids (EETs) and HETEs on cardiovascular health (Nayeem, 2018). Reviewed literature describes the cardioprotective effects of EETs through inducing hyperpolarisation of vascular smooth muscle cells and inducing vasodilation in vascular beds which may contribute to positive associations between EETs and decrease CVD risk in humans (Campbell et al., 1996; Nayeem, 2018; Nayeem et al., 2009). Opposing effects are reported for AA‐derived HETEs which induce secretion of vascular endothelial growth factors, negatively impacting vascular tone which may contribute to the positive association of HETEs and hypertension (Honda et al., 1999; Nakao et al., 1982) and acute myocardial infarction in patients with coronary artery disease (Huang et al., 2020). In addition, AA‐derived HETEs have been shown to induce the migration and chemotaxis of leukocytes further contributing to their role in cardiac dysfunction (Goetzl & Pickett, 1980; Goldman et al., 1983; Nayeem, 2018).

Evidence describing splenic and cardiac oxylipins in response to cardiac events that may highlight mechanisms of crosstalk within the cardiosplenic axis is starting to emerge. Following myocardial infarction, LOXs expressed in splenic macrophages convert fatty acids to SPMs (Halade et al., 2018; Swirski et al., 2009), and these splenic leukocytes migrate to the site of ischemia following cardiac injury to aid cardiac repair (Halade et al., 2018). 12/15‐LOX has most notably been described as a key mechanism of cardiac repair and survival following cardiac injury (Halade et al., 2018; Kain et al., 2018, 2024). Using an M1‐like macrophage‐specific 12/15‐LOX knockout (KO) murine model of heart failure, Kain et al. (2018) recently described reduced cardiac (left ventricle) SPMs in response to heart failure in 12/15‐LOX KO mice in comparison to wild‐type mice (8% of all oxylipins vs. 17%), with a reduction of D‐series resolvins, protectins and maresins by 70% in comparison to wild‐type mice (Kain et al., 2024). In addition, 12/15‐LOX KO mice exhibited a 30% increase in cardiac 11,12‐EET following myocardial infarction which improved survival in risk‐free mice post myocardial infarction (Kain et al., 2024). Prior to this, Kain et al. had reported lower splenic 12‐ and 15‐HETE in 12//15‐LOX KO mice alongside increased levels of splenic 5,6‐, 8,9‐ and 11,12‐EET in these animals following myocardial infarction (Kain et al., 2018). Coinciding with increased EET synthesis was reduced expression of splenic and cardiac *Ephx2* encoding soluble epoxide hydrolase, responsible for the degradation of EETs. In addition, an increase in EPA‐derived oxylipins 5‐, 8‐ and 9‐HEPE which have resolving actions were increased in 12/15‐LOX KO mice in comparison to wild type following myocardial infarction (Kain et al., 2018). These studies suggest 12/15‐LOX deletion shifts metabolism of AA towards EETs in a cardiosplenic manner which has reparative actions following heart failure (Kain et al., 2018, 2024).

There is also evidence for the role of oxylipin signalling in regulating immune cell communication of the cardiosplenic axis and cardiac health. Specific M1‐like macrophage deletion of 12/15‐LOX shifted neutrophil polarisation to a pro‐resolving phenotype and resulted in greater populations of Ly6C^lo^ CD206+ macrophages of a resolving phenotype in mice following myocardial infarction (Kain et al., 2018). More recently, Kain et al. further describe increased populations of splenic and cardiac (left ventricle) F4/80 Ly6C^lo^ macrophages, CD4+ cells—specifically CD4 + Foxp3+ regulatory T cells, and neutrophils in 12/15‐LOX KO mice in comparison to wild‐type mice in a model of chronic heart failure (Kain et al., 2024). Regulation of cardiosplenic immune cell communication by oxylipin signalling is further supported by reports of lipoxin receptor (ALX) deletion resulting in the expansion of splenic CCR2+ cell (macrophage) population with a similar trend observed in the heart (Tourki et al., 2020). Activation of leukocytes in the spleen and heart coincided with upregulated expression of genes encoding trafficking and migration supporting chemokines within C‐C motif chemokine ligand (CCL) and TNF subfamilies in the heart (Tourki et al., 2020). Furthermore, activation of splenic leukocytes increased the expression of genes associated with extracellular matrix deposition and degradation in the heart (Tourki et al., 2020).

These reports highlight emerging mechanisms by which oxylipin synthesis and signalling pathways regulate the cardiosplenic axis, and their potential to support cardiovascular health and improve outcomes following events associated with cardiovascular disease.

### Nutritional intervention to modulate splenic and cardiac oxylipins

LC n‐3 PUFAs are well‐described for their cardioprotective effects (Khan et al., 2021), but a lesser described, emerging mechanism of action is via oxylipin signalling. There are now several studies reporting changes to systemic oxylipins with LC n‐3 PUFAs (Dyall et al., 2022), but attribution to cardiac function and profiles of localised heart oxylipins remains less well‐described. One study adopting Fat‐1 transgenic mice as a model of ‘n‐3 PUFA protection’ due to increased endogenous EPA and DHA observed in the myocardium of these animals, details the protective effects of LC n‐3 PUFAs on cardiovascular health through oxylipin profile augmentation (Li, Tan, et al., 2021). In comparison to wild‐type (WT) mice, Fat‐1 mice had higher concentrations of EPA and DHA in the myocardium and lower levels of AA in addition to a lower n‐6/n‐3 ratio. Upon transverse aortic constriction (TAC), a method of inducing pressure overload within the heart mimicking hypertensive heart failure in humans, n‐6 oxylipins increased (15‐HETrE, 12‐keto‐LTB4, 12‐HHT, 9‐HETE and 6‐keto‐PGE1α) (Li, Tan, et al., 2021). However, in ‘n‐3 PUFA protected’ Fat‐1 mice, 12‐HHT, 9‐HETE, 12‐keto‐LTB4 and 6‐keto‐PGE1α were reduced following TAC in comparison to WT mice (Li, Tan, et al., 2021), highlighting the important role of LC n‐3 PUFA in regulating inflammation by oxylipins following cardiac events.

The regulation of splenic and cardiac oxylipin profiles and responses to cardiac events are not reported; however, augmentation of splenic oxylipins is observed with n‐3 PUFA feeding. Rats fed alpha‐linolenic acid (ALA), EPA or DHA exhibited decreases in AA‐derived splenic oxylipins and increases in ALA, EPA and DHA‐derived oxylipins (Pauls, Ragheb, et al., 2020). Feeding with high dose ALA, EPA or DHA (replacing 3 g of dietary MUFA with either ALA, EPA or DHA to provide a total of 10 g fatty acids/100 g of diet) decreased HETEs in both male and female rats but decreased several prostaglandins in female rats only (Pauls, Ragheb, et al., 2020). Feeding with EPA and DHA increased concentrations of HEPEs and HDHAs in both male and female rats, and the SPM protectin D1 (PD1) in male rats only (Pauls, Ragheb, et al., 2020). This highlights nutritional intervention with lipids as a route to modulating spleen oxylipin profile which considering the evidence reviewed for the cardiosplenic axis, may have benefits on cardiovascular outcomes, but that some of these effects may be sex dependent.

Differences in levels of plasma oxylipins by sex have also been reported in humans (Gabbs et al., 2021; Pauls, Du, et al., 2020). Pauls et al. describe levels of several plasma oxylipins to be habitually highest in young females in a cohort of men and women (young females *n* = 26, older females (postmenopausal) *n* = 31, older males *n* = 21) (Pauls, Du, et al., 2020; Pauls, Ragheb, et al., 2020). Of 38 n‐6 PUFA‐derived oxylipins and 25 n‐3 PUFA‐derived metabolites that were measured, 9 and 14 were significantly greater in young females, respectively, including 12‐HEPE, and 4‐, 8‐, 11‐, 13‐, 14‐. 16‐ and 20‐HDHA. However, not all of these differences could be attributed to sex hormones, specifically oestradiol levels. Oestradiol was associated with few oxylipins and the magnitude of the associations was weaker than expected given the significant difference between young females, older females and males. Despite this, oestradiol concentrations were positively associated with 12‐LOX derived oxylipins, 12‐HETE, 12‐HEPE and 14‐HDHA which were more than threefold higher in young females (Pauls, Du, et al., 2020).

**FIGURE 1:**
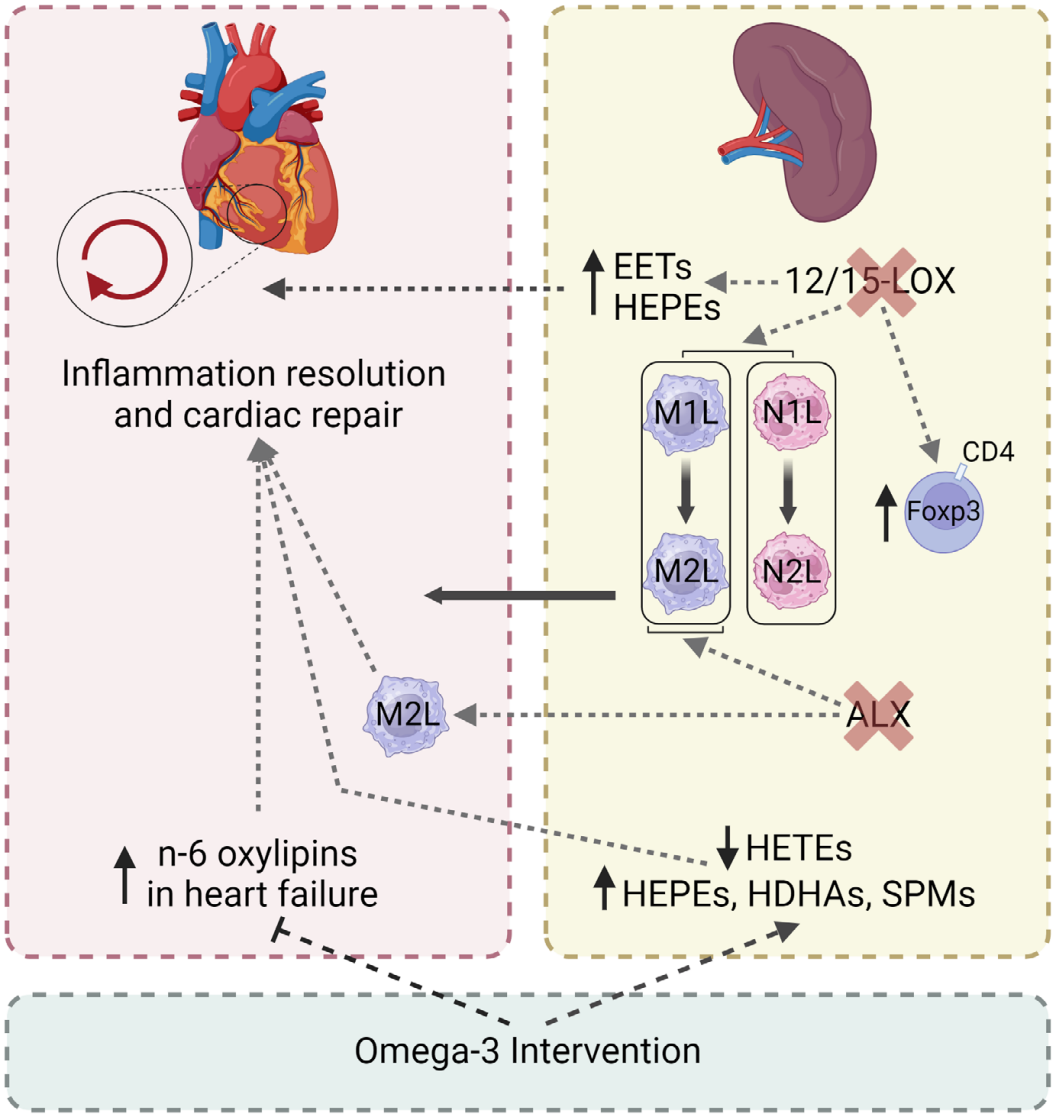
Regulation of the cardiosplenic axis by oxylipins and effects of dietary omega‐3 intervention. Oxylipins play a role in cardiac remodelling and repair after injury in a cardiosplenic manner. There is evidence for the role of splenic oxylipin synthesis and signalling pathways (demonstrated here through deletion of 12/15‐LOX and ALX) regulating immune cell polarisation, inflammation resolution and cardiac repair following cardiac injury. Furthermore, dietary omega‐3 intervention has been seen to decrease synthesis of AA‐derived HETEs and increase EPA and DHA‐derived oxylipins (HEPEs, HDHAs and PD1, respectively). These oxylipins have been observed to have a reparative effect following myocardial infarction and in acute and chronic heart failure. 12/15‐LOX, 12/15 lipoxygenase; ALX, lipoxin receptor; EETs, epoxy‐eicosatrienoic acids; HDHAs, hydroxy‐docosahexaenoic acids; HEPEs, hydroxy‐eicosapentaenoic acids; HETEs, hydroxy‐eicosatetraenoic acids; M1L, M1‐like pro‐inflammatory macrophage phenotype; M2L, M2‐like pro‐resolving macrophage phenotype; N1L, N1‐like pro‐inflammatory neutrophil phenotype; N2L, N2‐like pro‐resolving neutrophil phenotype; n‐6, omega‐6; SPMs, specialised pro‐resolving mediators. Created in BioRender. Fisk (2024) BioRender.com/q23l517.

Differences in response to intervention are also reported by sex in humans. Gabbs et al. reported faster accumulation and greater increases in plasma oxylipins in response to daily high doses of ALA (4.2 g) or DHA (4.3 g) over 6 weeks in 12 young adult participants (6 males, 6 females). Sixteen of 62 measured oxylipins were reported to increase more in female participants than male. These included 5 of 10 linoleic acid (LA) derived oxylipins, 1 of 3 dihomo gamma linoleic acid (DGLA), 3 of 30 AA, 1 of 5 ALA, 1 of 6 EPA and 5 of 12 DHA‐derived oxylipins. DHA intervention increased plasma DHA more than twofold which did not differ by sex, although there was a significantly greater increase in hydroxy‐DHA oxylipins including 8‐, 11‐, 14‐ and 20‐HDHA in female participants. There was no consideration of sex hormones in this study (Gabbs et al., 2021). These two studies highlight the effect of sex on both habitual levels of oxylipins and oxylipin response, particularly DHA oxylipins, to LC n‐3 PUFA intervention and the need for consideration in future analyses. Further investigation of the effects of sex hormones and their contribution to the sex differences in response to LC n‐3 PUFA intervention is needed to advance understanding and tailor therapeutic intervention to ensure health benefits across populations. Figure 1 provides a graphical summary depicting the regulation of the cardiosplenic axis by oxylipins, and effects of dietary omega‐3 intervention.

## GUT‐LINKED AXES

Knowledge and understanding of how the gut microbiome can impact host homeostasis and health is continuing to expand with evidence supporting the role of host microbiome on brain, liver and lung function and health, and energy homeostasis (Duca et al., 2014; Saxami et al., 2023; Zhang et al., 2020). Changes in the intestinal microbiome have been observed in obesity (Aoun et al., 2020; Liu et al., 2021), metabolic‐dysfunction associated steatotic liver disease (MASLD) (Cai et al., 2024) and in pulmonary conditions such as asthma and chronic obstructive pulmonary disease (COPD) (Zhang et al., 2020). The intestinal microbiota can impact inflammation, which is a key underlying feature of the diseases mentioned, through modulating communication via the central nervous system (CNS), enteral nervous system (ENS) and the autonomic nervous system (ANS), as well as through modulation of neurotransmitters, cytokines, short chain fatty acids (SCFAs) and bacterial metabolites (Saxami et al., 2023). In addition, there is emerging evidence from animal models highlighting communication by the intestinal microbiota through modulation of oxylipin signalling. Dietary and antibiotic‐induced changes in intestinal microbiota were associated with changes to circulating oxylipins in rats (Avila‐Roman et al., 2021). Relationships between dietary lipids, microbial diversity and microbial metabolites including oxylipins have been demonstrated (Avila‐Roman et al., 2021; Miyamoto et al., 2019; Roussel et al., 2024), suggesting inflammation can be altered through changes to the diversity and complexity of intestinal microbiota (Cani & Van Hul, 2024; Liu et al., 2021).

A study in human adults (*n* = 80) reported significant associations between plasma oxylipins and faecal microbiota (Xu et al., 2022). Omega‐3 derived oxylipins 8‐HDHA, 13‐HDHA and 19,20‐DIHDPE were positively associated with the relative abundance of *Clostridium cluster IV* genus (*Firmicutes* phylum) and 5‐HEPE, 4‐HDHA and 19,20‐HDPE were negatively associated with *Sutterella* genus (*Proteobacteria* phylum). Omega‐6 derived oxylipins (combined total of detected LA, DGLA and AA‐derived oxylipins) were negatively associated with the relative abundance of *Acidaminococcus* and *Phascolarctobacterium* genera (*Firmicutes* phyla), *Sutterella*, *Succinivibrio* and *Gemmiger* genera (*Proteobacteria* phylum) (Xu et al., 2022). These observations in humans, highlighting the relationship between the gut microbiome and plasma oxylipins, support the potential for modulation of oxylipins and associated inflammatory signalling through modification of gut microbiota.

In addition to discussing several gut‐linked axes, here we will discuss evidence for the modification of gut microbiota, oxylipin profiles, their cross‐regulation and potential impact on inflammatory signalling and communication across multiple organs.

### Gut–brain axis

There is bidirectional communication between the intestinal microbiota and the brain (Sandhu et al., 2017; Saxami et al., 2023). This occurs via the sympathetic and parasympathetic branches of the ANS, ENS, the hypothalamic–pituitary–adrenal axis of the endocrine system and the immune system (Saxami et al., 2023). In addition, enteroendocrine hormones can modify host physiology regulating appetite, satiety, nutrient absorption and behaviour (Saxami et al., 2023). Within this complex communication system, hormonal, immune and neural signals, bacterial metabolites such as SCFAs, and immune mediators such as chemokines, are the main routes by which communication is established (Saxami et al., 2023).

As briefly outlined, oxylipins are modulated by the gut microbiota and are an emerging route by which communication is established (Avila‐Roman et al., 2021; Miyamoto et al., 2019; Roussel et al., 2024). Oxylipins play a role in ENS signalling (Mantel et al., 2023); AA‐derived oxylipins produced at the mucosal and submucosal level stimulate enteric neurons in the gastrointestinal tract through IL‐1β mediated signalling, and prostaglandins E2 and D2 prolong depolarisation of enteric neurons in guinea pigs (Dekkers et al., 1997; Frieling et al., 1994; Kelles et al., 2002). In addition, leukotrienes C4, D4 and E4 were shown to slow depolarisation of myenteric neurons in the small intestine further evidencing the potential role of oxylipins in enteric neuron excitability and signalling. The ENS may also be a source of oxylipins itself; secreted phospholipase A2 (PLA2) has been detected in the myenteric ganglia of the small intestine and neuronal fibres of the stomach (Masuda et al., 2005; Surrel et al., 2009), COX‐1 and COX‐2 enzymes have been found in human myenteric ganglia (Bernardini et al., 2006; Fornai et al., 2005) and 15‐LOX is expressed in human enteric glial cells (Pochard et al., 2016), suggesting ability for oxylipin production.

Gut dysbiosis has been reported in several neurological diseases, further evidencing a relationship between the intestinal microbiome and the brain. Changes to bacterial genera and phyla are seen in individuals living with Parkinson's disease (Gerhardt & Mohajeri, 2018; Sun & Qin, 2018), amyotrophic lateral sclerosis, multiple sclerosis and autism spectrum disorder (Saxami et al., 2023; Varesi et al., 2022). Therefore, communication by microbiota including differences in oxylipin signalling may contribute to altered inflammatory signalling in these conditions. This is an emerging area for future study.

In addition, differences in oxylipin profiles of individuals living with Alzheimer's disease (AD) have been reported. In comparison to healthy controls, lower levels of plasma EPA and DHA‐derived oxylipins (5‐, 9‐ and 12‐HEPE, and 4‐, and 14‐HDHA) were observed (Borkowski et al., 2021). Differences in plasma oxylipins were also observed in humans with mild cognitive impairment with higher 12,13‐dihydroxy‐octadecenoic acid (DiHOME)/12,13‐epoxyoctadecaenoic acid (EpOME), 14,15‐ and 17,18‐DiHETE and 19,20‐DiHDPA in fasted plasma, and lower 15‐HEPE, 4‐HDHA and 14‐HDHA in non‐fasted plasma associated with worse processing speed (Borkowski, Taha, et al., 2021). It is important to note that changes in oxylipin levels in AD or other cognitive impairments do not infer causality, which will require mechanistic studies at the molecular level.

Whilst associations have not been made between oxylipins and the gut microbiome in cognitive and neurological disorders, changes to the gut microbiome are reported (Liang et al., 2024) which, in light of emerging evidence describing cross‐regulation between these in humans, may contribute to differences observed in oxylipins. In a recent review, Liang et al. comprehensively discuss literature describing changes to the gut microbiota in impaired cognition and AD (Liang et al., 2024). A reduction in bacterial diversity and differences in bacterial phyla were noted across several studies of impaired cognition and AD; in general, reduced *Firmicutes* and increased *Bacteroidetes* phyla were described (Liang et al., 2024). There are some inconsistencies in current evidence as a few studies report a decreased abundance of *Bacteroidetes* (Zhuang et al., 2018) highlighting the need for further research in this area. Mapping the lipidome inclusive of oxylipins in addition to the gut microbiome may further our understanding of the impacts of these changes and variation observed amongst individuals.

### Adipose–gut–brain axis

There is evidence for crosstalk between the gut–brain axis and the adipose tissue which plays a key role in regulating metabolism and energy homeostasis. The intestinal microbiome and communication with the brain can regulate the lipid storage capacity of the white adipose tissue (WAT) through modulating lipid oxidation. Effects of the microbiome on lipid oxidation have been reported in studies that transplanted faeces from individuals who had undergone bariatric surgery to germ‐free mice, which induced a reduction in fat deposition and decreased use of carbohydrate as fuel (indicated by reduced respiratory quotient) (Tremaroli et al., 2015). Furthermore, transplantation of *Bifidobacterium* species to germ‐free mice inhibits fasting‐induced adipocyte factor which is a lipoprotein lipase inhibitor linked to rapid hepatic lipogenesis and insulin resistance (Wang et al., 2021). This links the role of the microbiota to reduction of adiposity, metabolic syndrome and changes in lipid metabolism.

#### Mechanisms of crosstalk between gut and adipose tissue

Endocrine signalling between the WAT and the gut–brain axis further contributes to regulating energy storage and obesity (Cheng & Liu, 2020; Ya et al., 2020). Leptin, secreted by the adipose tissue in proportion to mass, signals via the CNS to regulate lipid metabolism (Caron et al., 2018; Shaikh et al., 2024). Leptin secretion has been shown to be affected by changes in intestinal microbiota in which reversal of leptin resistance following high‐fat diet was observed in mice, alongside increased expression of thermogenic and lipid metabolism‐related genes in the adipose tissue (Cheng & Liu, 2020). In addition, the adipokines adiponectin and resistin modulate changes in microbiota. Use of antibiotics in diet‐induced obese mice resulted in decreased mRNA expression of adiponectin and resistin alongside increased expression of peroxisome proliferator‐activated receptor‐alpha (*PPARA*), peroxisome proliferator‐activated receptor‐gamma coactivator‐1alpha (*PPARGC1A*) and adipose triglyceride lipase (*PNPLA2*) which are associated with fat oxidation and thermogenesis (Ya et al., 2020). Of important note is the regulation of such adipokines in obesity. Increased leptin and resistin and reduced adiponectin are observed in obesity and are associated with inflammatory status (Zorena et al., 2020). In addition, modification of the intestinal microbiome richness and phylogenetic diversity is observed in obesity, as reviewed elsewhere (Angelini et al., 2024), with several reports noting an increased *Firmicutes*/*Bacteroides* ratio (Angelini et al., 2024), which may link to the observed dysregulation in adipokines.

#### Role of oxylipins in gut–adipose crosstalk

In addition to adipokines, there are several reports of changes to oxylipins and endocannabinoids in human obesity (Fisk et al., 2021, 2022; Hateley et al., 2024; Lopez‐Vicario et al., 2019). Dysregulation of oxylipins, responses to LC n‐3 PUFA and mechanisms of associated immune dysfunction in obesity are eloquently described elsewhere (Shaikh et al., 2024; De Bus et al., 2019) and will not be further described in this review. There is evidence of a reduction of DHA‐derived oxylipins in leukocytes (Lopez‐Vicario et al., 2019), plasma (Lopez‐Vicario et al., 2019) and WAT (Fisk et al., 2022), reduction of resolvins in WAT (Fisk et al., 2022), reduction of lipoxin B4 in leukocytes (Lopez‐Vicario et al., 2019) and WAT (Fisk et al., 2022), reduction of dihydroxy‐eicosatrienoic acids (DHETs) derived from AA and EpOMEs and DiHOMEs derived from linoleic acid (LA) in omental WAT (Hateley et al., 2024), and an increase in the endocannabinoid arachidonoyl ethanolamide (anandamide, AEA) from AA and the endocannabinoid‐like molecule eicosapentaenoyl ethanolamide (EPEA) from EPA in WAT (Fisk et al., 2022), in individuals living with obesity. Dysregulation of resolving oxylipins is often observed alongside markers of an increased inflammatory environment but mechanistic links between the gut microbiome, oxylipin signalling and inflammation in obesity have not been described and are worthy of focus. A good starting point would be with the use of rodent models as the microbiome will be easier to manipulate than in humans and will allow for more causal studies on oxylipins and the microbiota.

The role of oxylipin signalling in communication between the adipose tissue and the gut–brain axis is not understood. However, evidence for the role of the endocannabinoid system is stronger. Endocannabinoids have a role in maintaining intestinal homeostasis by regulating intestinal permeability and inflammatory response in the gut (Srivastava et al., 2022). Animal models have described communication between adipose, gut and brain through the modulation of endocannabinoid tone and intestinal microbiota by high‐fat diet feeding, resulting in altered intestinal permeability and dysregulated adipogenesis (Muccioli, 2010). In addition, the deletion of N‐acyl phosphatidyl ethanolamide phospholipase D (NAPE‐PLD) which is required for the synthesis of N‐acyl ethanolamides (endocannabinoids) in white adipocytes resulted in augmented intestinal microbiome (Everard et al., 2019). Furthermore, changes to the endocannabinoid profile were observed in mice treated with antibiotics which augmented intestinal microbiome (Guida et al., 2018).

Therefore, links between gut microbiota and oxylipin signalling may be implicated in obesity‐associated inflammation and may be a potential route for therapeutic intervention to improve outcomes in this group of individuals.

### Gut–liver axis

Changes to the gut microbiome have been observed in individuals with liver conditions; dysbiosis varies with aetiology of liver disease and is confounded by comorbidities including obesity and metabolic syndrome (Hsu & Schnabl, 2023). This has been comprehensively reviewed elsewhere (Hsu & Schnabl, 2023). Here, we will focus on mechanisms of communication between the gut and liver and implications in liver disease.

#### Mechanisms of crosstalk between the gut and liver

The gut and liver share a key route of connection and communication via the portal and arterial circulation. The liver encounters enterally absorbed nutrients and microbial metabolites from venous blood draining from the small and large intestines into the portal vein (Hsu & Schnabl, 2023). In addition, the liver can communicate to the gut through release of hepatic metabolites and molecules including the secretion of bile which contains lipids (cholesterol and phospholipids) and bile acids, as well as secretion of proteins, antimicrobial molecules and immunoglobulin‐A (IgA), into the small intestine (Hsu & Schnabl, 2023). Bile acids facilitate digestion and absorption of lipids and lipid‐soluble vitamins which can contribute to microbiome composition. In addition, bile acids exert bacteriostatic effects via their detergent‐like properties and through activation of farnesoid X receptors (FXR) which stimulate production of antimicrobial molecules (Inagaki et al., 2006). Agonism of FXR and suppression of bile acid synthesis results in proliferation of gram‐positive bacteria in the small intestine, further evidencing the role of hepatic bile acids in the modulation of the gut microbiome (Friedman et al., 2018). In addition, gut bacteria deconjugate and dehydroxylate liver‐derived bile acids reducing their reabsorption in the ileum where they can activate FXR target genes and affect metabolism (Fuchs & Trauner, 2022). For example, bile acid activation of fibroblast growth factors 15‐ and −19 regulates insulin sensitivity and hepatic glycogen synthesis (Inagaki et al., 2005).

Individuals living with MASLD or non‐alcoholic steatohepatitis (NASH) have higher levels of serum and total bile acids, and increased concentrations of secondary (dehydroxylated) bile acids in comparison to primary hepatic bile acids (Hsu & Schnabl, 2023). In addition, changes to gut microbiota are observed in these conditions, as reviewed elsewhere (Hsu & Schnabl, 2023). The bidirectional communication described between bile acids and gut microbiota is complex, and as a result, it has not been ascertained whether changes to bile acids or microbiota are cause or consequence.

In addition to bile acids, other well‐described microbial metabolites, including SCFAs, lipopolysaccharide (LPS), ethanol and choline metabolites, are delivered from the intestine to the liver by the portal circulation and have effects on liver metabolism, inflammation and immune tolerance (Anand & Mande, 2022). Intestinal bacterial sphingolipids translocating to the liver reduce hepatic lipid accumulation by increasing beta‐oxidation in mice (Le et al., 2022). In addition, transfer of LPS to the liver is key for immune tolerance; hepatic sinusoidal endothelial cells sense microorganisms and metabolites and signal Kupffer cell localisation for phagocytosis of transferred microbial products and viable bacteria (Hsu & Schnabl, 2023). Furthermore, microbiota‐derived SCFAs have both beneficial and negative effects in the liver; for example, increased acetate is associated with increased hepatic triglyceride accumulation, whereas butyrate has been shown to activate AMP‐activated protein kinase to reduce inflammation and modulate lipid and glucose metabolism to reduce insulin resistance (Anand & Mande, 2022).

#### Regulation of liver function by oxylipins and endocannabinoids

Endocannabinoids and endocannabinoid‐like signalling molecules have also been shown to play a role in regulating liver function. These molecules signal via G‐protein coupled receptors (cannabinoid 1 [CB1] and cannabinoid 2 [CB2] receptors) expressed in the liver (Bazwinsky‐Wutschke et al., 2019). CB1 is the main receptor observed to be expressed in liver cells (hepatocytes, stellate cells and sinusoidal epithelial cells), whilst CB2 is expressed in resident Kupffer cells (Bazwinsky‐Wutschke et al., 2019). Omega‐6 fatty acid containing endocannabinoids, AEA and 2‐arachidonoylglycerol (2‐AG), bind to CB1 in the liver to promote hepatic lipogenesis and steatosis (Bazwinsky‐Wutschke et al., 2019). In addition, CB1 activation promotes fibrogenesis whilst antagonism of CB1 can improve glucose tolerance and insulin resistance in diet‐induced obese mice (Bajzer et al., 2011). Supplementation of individuals living with obesity and new diagnosis of MASLD with oleoylethanolamide (OEA) alongside calorie restriction, resulted in greater mRNA expression of PPAR‐ α (observed to decrease with NASH development) and uncoupling protein (UCP)‐1 and UCP2 (involved in mitochondrial fatty acid β‐oxidation) in PBMCs. In addition, OEA supplementation resulted in greater amelioration of liver health markers alanine aminotransferase (ALT), aspartate aminotransferase (AST) (which increase with liver injury) and ALT/AST ratio than the placebo plus calorie restriction group (Tutunchi et al., 2020). In addition to decreasing serum triglycerides, liver steatosis was reduced in both groups but there was a trend for greater reduction with OEA supplementation (Tutunchi et al., 2020). Furthermore, OEA supplementation in addition to calorie restriction resulted in greater reductions in insulin and HOMA‐IR in comparison with the placebo plus calorie restriction group (Tutunchi et al., 2020).

Further evidence for the role of endocannabinoids in liver function has been observed in a murine study. Deletion of intestinal epithelial cell NAPE‐PLD in mice exacerbated steatosis upon high‐fat diet feeding in comparison to wild‐type mice (Everard et al., 2019). Greater hepatic lipid droplet size, accumulation of triglycerides and total hepatic lipid content were observed in NAPE‐PLD null mice in comparison to wild‐type mice (Everard et al., 2019). In addition, elevation of serum ALT levels were observed with NAPE‐PLD deletion (Everard et al., 2019). Consideration of previously discussed evidence describing changes to endocannabinoids with antibiotic use (Guida et al., 2018), there is support for the modulation of liver function through modification of endocannabinoids and gut microbiota but further evidence is required.

In addition to endocannabinoids, there is evidence for the role of oxylipins in regulating liver function. For example, mice fed DHA exhibited reduced hepatic COX‐2 expression and prostaglandin‐E2 (PGE2) levels and increased hepatic formation of 17‐HDHA and protectin D1 (Gonzalez‐Periz et al., 2006). In vitro incubation of hepatocytes with DHA reduced DNA damage and oxidative stress upon exposure to hydrogen peroxide (Gonzalez‐Periz et al., 2006), and synthetic 17‐HDHA was observed to decrease 5‐lipoxygenase (5LOX) expression in hepatic macrophages (Gonzalez‐Periz et al., 2006; Rius et al., 2014). Furthermore, the use of resolvins has been shown to improve liver inflammation; resolvin D1 (RvD1) administration in combination with calorie restriction reduced liver macrophage infiltration and reprogrammed inflammatory M1‐like macrophages to an M2‐like pro‐resolving phenotype, and reduced hepatic steatosis and insulin resistance in mice (Rius et al., 2014). Resolvin E1 also improves the hepatic transcriptome, which is dependent on the G‐protein coupled receptor ERV1/ChemR23 (Al‐Shaer et al., 2021). Overexpression of myeloid ERV1 also improved hepatic inflammatory outcomes in addition to glucose tolerance (Sima et al., 2017).

In a further feeding study using an LDL‐receptor deficient mouse model, supplementing western diet with LC n‐3 PUFA lowered hepatic AA and AA‐derived oxylipins, and increased hepatic EPA, DHA and derived oxylipins in membrane lipids (Garcia‐Jaramillo et al., 2019). This included increasing epoxy‐eicosatetraenoic acids (EpETEs), dihydroxy‐eicosatetraenoic acids (DiHETEs), epoxy‐docosapentaenoic acids (EpDPEs), dihydroxy‐docosapentaenoic acids (DiHDPEs) and protectin D1 (Garcia‐Jaramillo et al., 2019). In addition, C20‐22 omega‐3 derived CYP2C and CYP2J pathways oxylipins were inversely associated with NASH markers of inflammation and hepatic fibrosis (Garcia‐Jaramillo et al., 2019).

#### Role of oxylipins in gut–liver crosstalk

What is not so well‐known is the role of oxylipins in communication between the gut and the liver. In humans, there is evidence of augmented oxylipin profiles with liver disease with increased hepatic 14,15‐EET/DHET ratio in individuals with type‐2 diabetes and increased hepatic 12,13‐EpOME/DiHOME ratio, a marker of WAT inflammation with steatosis, in the liver in individuals living with obesity (Hateley et al., 2024). Correlations between these oxylipins and modified gut microbiota in liver diseases are not reported.

**FIGURE 2:**
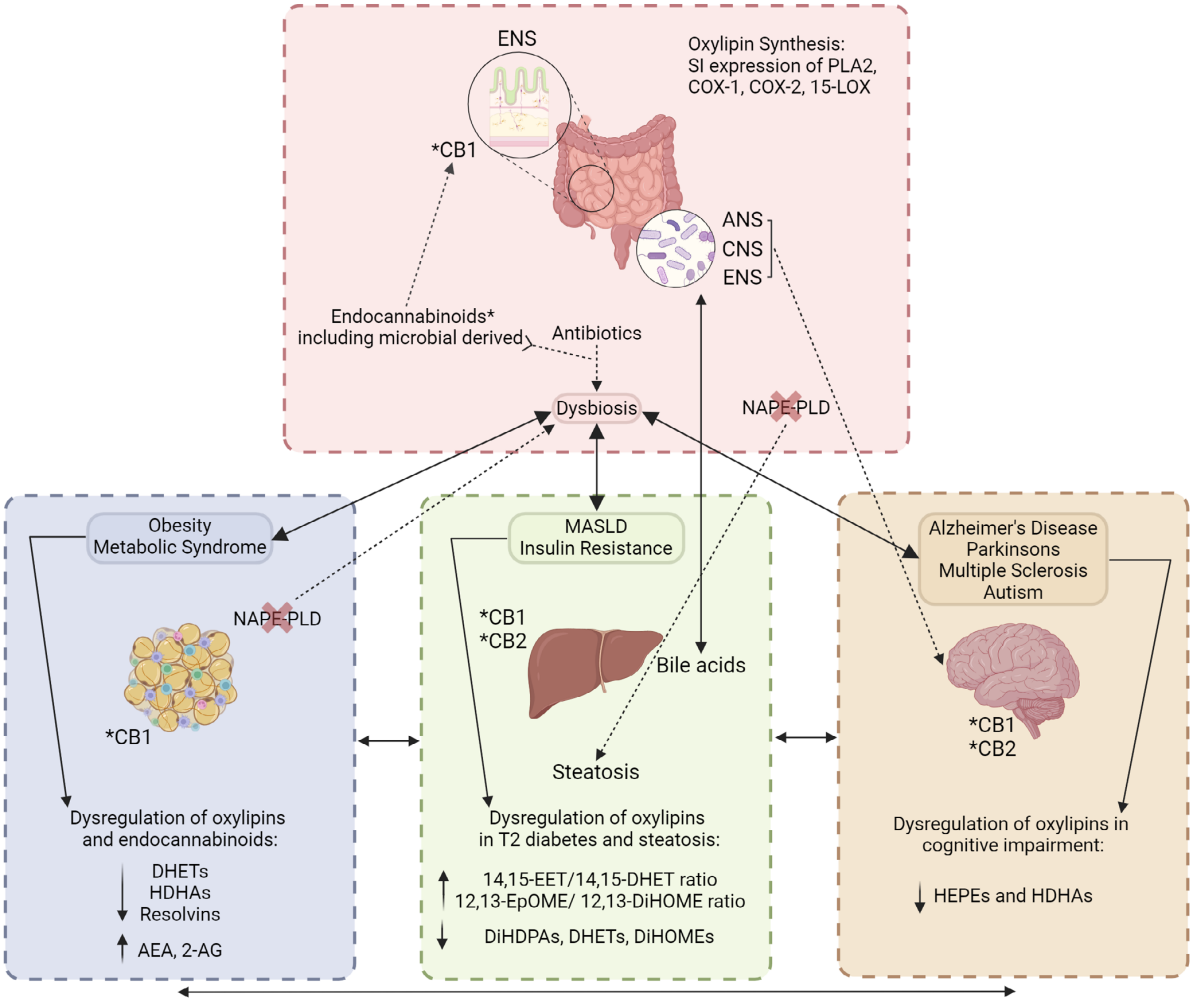
Regulation of gut‐linked axis and organ crosstalk by microbiota and oxylipins. There is evidence for augmented microbiome and oxylipin profiles in a range of conditions spanning multiple organs; for example, obesity and metabolic syndrome, MASLD and neurological conditions such as as Alzheimer's and Parkinson's diseases. Associations between these profiles are not understood but there is evidence of expression of enzymes involved in oxylipin synthesis in the small intestine and for the role of the endocannabinoid system in regulating intestinal microbiome and whole‐body metabolism. Cannabinoid receptors are expressed in the small intestine, adipose tissue, liver and the brain, highlighting a key route of organ crosstalk for the regulation of inflammation and metabolism. Deletion of adipose NAPE‐PLD results in augmented intestinal microbiome, endocannabinoids and hepatic steatosis. 12,13‐DiHOME, 12, 13‐dihydroxy‐octadecamonoenoic acid; 12,13‐EpOME, epoxy‐octadecamonoenoic acid; 14,15‐DHET, 14,15‐dihydroxy‐eicosatrienoic acid; 14,15‐EET, 14,15‐epoxy‐eicosatrienoic acid; 15‐LOX, 15‐lipoxygenase; AEA, arachidonyl ethanolamide, anandamide; ANS, autonomic nervous system; CB1, cannabinoid receptor 1; CB2, cannabinoid receptor 2; CNS, central nervous system, COX‐1, cyclooxygenase‐1; COX‐2, cyclooxygenase‐2; DHETs, dihydroxy‐eicosatrienoic acids; DiHDPAs, dihydroxy‐docosapentaenoic acids; DiHOMEs, dihydroxy‐octadecamonoenoic acids; ENS, enteric nervous system; HDHAs, hydroxy‐docosahexaenoic acids; HEPEs, hydroxy‐eicosapentaenoic acids; NAPE‐PLD, N‐acyl phosphatidylethanolamine‐phospholipase D; PLA2, phospholipase‐A2. Created in BioRender. Fisk (2024) BioRender.com/k19l964.

One clinical study administered a symbiotic (fructo‐oligosaccharides plus *Bifidobacterium animalis—lactia BB‐12*) over 10–14 months to patients with MASLD and reported significantly altered faecal microbiome but that this was not associated with changes to liver fat content or markers of liver fibrosis (Scorletti et al., 2020). This intervention did however increase microbial beta‐diversity, increasing abundance of *Bifidobacterium* and *Faecalibacterium* species and reducing the abundance of *Oscillibacter* and *Alistipes* species (Scorletti et al., 2020). What was not reported but would be of great interest is if there were changes to oxylipin profiles with the intervention which may highlight changes to inflammatory signalling in the absence of change to liver fat or fibrosis. Exploration of the cross‐regulation of the microbiome and oxylipin signalling, both reported to be modified in liver disease, is lacking and is of great interest with potential to improve outcomes in this group of individuals. Figure 2 provides a graphical summary depicting the regulation of gut‐linked axis and organ crosstalk by microbiota and oxylipins.

## MODIFICATION OF MICROBIOME AND OXYLIPINS BY NUTRITIONAL INTERVENTION

The resolving capabilities of oxylipins derived from LC n‐3 PUFAs have sparked interest in intervention studies using EPA and DHA aiming to lower pro‐inflammatory oxylipins and increase anti‐inflammatory and pro‐resolving oxylipins, ultimately returning damaged tissue to homeostasis. Here we discuss updates to research utilising feeding studies to modify oxylipins and gut microbiome. As a point of reference for dietary fatty acid doses discussed below, daily intakes of EPA + DHA are globally recommended between a minimum of 250–500 mg/day (Troesch et al., 2020). In addition, there is the suggestion that RBC levels of EPA + DHA should be 8% or above to confer cardioprotection, although the majority of the world's population is generally below this value (Schuchardt et al., 2024).

In wild‐type and fat‐1 transgenic mice, an LC n‐3 PUFA enriched diet (1% EPA and 1% DHA as ethyl esters/day for 30 days) decreased the n‐6/n‐3 ratio in whole blood, plasma and tissues including brain, spleen, liver, kidney and colon (Ostermann et al., 2017). This was accompanied by changes to oxylipins in plasma, brain and colon. LC n‐3 PUFA feeding resulted in decreased plasma PGE2, EpETrEs, DiHETrEs and HETEs and increased HEPEs, EpDPAs, DiHDPAs and HDHAs (Ostermann et al., 2017). In the brain, a range of prostaglandins (PGs), thromboxane‐2 (TXB2), 11‐ and 15‐HETE and several DiHETEs were decreased, and several HDHAs including 14‐HDHA were increased with LC n‐3 PUFA feeding (Ostermann et al., 2017). In addition, there were marked changes to the oxylipin profile of the colon with LC n‐3 PUFA feeding. There was a reduction in several PGs, EpETrEs, DiHETEs, DiHETrEs, HETEs, leukotrienes and isoprostanes, and increased levels of EpETEs, HEPEs, EpDPEs, DiHDPEs and HDHAs (Ostermann et al., 2017).

In a murine model, feeding with LC n‐3 PUFA (1% EPA and 1% DHA as ethyl esters/day for 2 weeks) increased several HEPEs, EPA‐derived PGs, EpDPEs and HDHAs in plasma and several tissues including liver, kidney, spleen, lung, small intestine, muscle, WAT, heart and brain (EPA derived only) (Naoe et al., 2019). There was no available data on the faecal microbiome of the animals in either study which would be of great interest to further understand the effects of modification of circulating and tissue specific, particularly colonic, oxylipins on the microbiome and cross‐regulation of these systems.

The effects of omega‐3 feeding on gut microbiota remain an emerging area of research with limited reports in humans. One study highlights a prebiotic effect of LC n‐3 PUFAs describing increases in *Bacteroides* spp. and *Coprococcus* spp. and significant decreases in *Collinsella* spp. which has been associated with fatty liver disease in response to daily 6‐week intervention of LC n‐3 PUFA (Vijay et al., 2021). LC n‐3 PUFA supplementation (165 mg of EPA, 110 mg DHA/day for 6 weeks) exhibited similar effects as inulin fibre, increasing the SCFAs iso‐butyrate and isovalerate (Vijay et al., 2021). *Coprococcus* bacteria was positively associated with isobutyric acid and negatively associated with serum triglyceride‐rich lipoproteins, very low‐density lipoprotein (VLDL) and VLDL‐TG (Vijay et al., 2021).

In addition to modulation of oxylipins by omega‐3 feeding, there is evidence for the therapeutic use of pro‐resolution oxylipins in gut health and inflammatory bowel diseases as reviewed elsewhere (Pascoal et al., 2022; Wallace, 2019). Evidence is limited in humans but a key example using a murine model of colitis highlights the protective role of resolvin E1 in gut inflammation (Arita et al., 2005). Administration of resolvin E1 was shown to decrease the severity of 2,4,6‐trinitrobenzene sulfonic acid‐induced colitis in mice, halting leukocyte infiltration and downregulating the expression of related pro‐inflammatory genes (TNF‐α, IL‐12 p40, inducible nitric oxide synthase and COX‐2) (Arita et al., 2005). This protective role of resolving oxylipins in bowel inflammation may be achieved through omega‐3 feeding aiming to promote resolvin synthesis or by therapeutic resolvin administration. This may have the potential to support augmentation of gut microbiome profiles but investigation in this area is required.

### Evidence for co‐regulation of microbiome and oxylipin profiles

As demonstrated by studies investigating either microbiome or oxylipins, investigation of the modification of both, and relationship between these systems is lacking. Emerging evidence for regulation of both gut microbiota and oxylipins has been described in mice in which feeding with PUFA‐derived oxylipins has been shown to alter gut microbiota, oxylipins and attenuate high‐fat diet (HFD) fed obesity and metabolic disturbance (Miyamoto et al., 2019). LA‐fed mice (1%/day for 12 weeks) exhibited significant production of the LA‐derived microbial metabolite 10‐hydroxy‐cis‐12‐octadecenoic acid (HYA) by *Lactobacillus salivarius* and *Lactobacillus gasseri* in 22 *Lactobacillus* strains (Miyamoto et al., 2019). In response to 12‐week supplementation with HYA itself (1%/day), mice exhibited increased *Lactobacillaceae* abundance, less weight gain on a HFD, lower WAT mass and reduced adipocyte size, and attenuation of HFD‐induced insulin resistance and impaired glucose tolerance (Miyamoto et al., 2019).

In addition, a recent study ‘feeding’ with buglossoides arvensis (ahiflower) oil which is rich in omega‐3 stearidonic acid (SDA 18:4, n‐3), reported significant changes to ileum and ascending colon luminal bacterial phyla (Roussel et al., 2024). This study used a mucosal simulator of the human intestinal microbial ecosystem (SHIME) which mimics the intestinal system from the stomach to ascending colon, in which an ileal microbial community was established using faecal microbiota collected from human donors (Roussel et al., 2024). Ahiflower oil (1200 mg, containing up to 21% SDA) was introduced to the SHIME stomach as a ‘feeding’ protocol over 14 days (Roussel et al., 2024). Ahiflower oil ‘feeding’ increased luminal *Bacteroida* genera *Bacteroides* and *Parabacteroides*, *Negativicutes* genera *Acidaminococcus* and *Gammaproteobacteria* genus *Escherichia*‐*Shigella*, and decreased abundance of *Clostridia* class bacteria (Roussel et al., 2024). It also modified mucus‐associated bacterial microbiota, increasing *Pseudomonas*, *Sellimonas intestinalis* and decreasing *Erysipelatoclostridium* (Roussel et al., 2024). In addition, Ahiflower oil ‘feeding’ resulted in changes to levels of the SCFA propionate, produced by *Bacteriodes* bacteria which was significantly associated with increased *Bacteroides* bacteria, and decreased the SCFAs acetate and butyrate which were associated with decreased *Clostridia* bacteria (Roussel et al., 2024). Furthermore, Ahiflower oil ‘feeding’ promoted active breakdown of SDA and synthesis of gut microbial endocannabinoid N‐stearidonoyl‐ethanolamide (SDEA) and the endocannabinoid‐like molecule commendamide (Roussel et al., 2024). There was a significant positive correlation between colonic SDEA and bacterial species, most notably *Butyricicoccus*, *Campylobacter* and *Phascolarctobacterium*, and associations between *Bacteroides* species and the *Bacteroides*‐derived endocannabinoid‐like mediator, commendamide (Roussel et al., 2024). Commendamide was not present in the stomach which was void of bacteria but increased in the ileum with Ahiflower oil ‘feeding’, highlighting the role of the intestinal microbiota in the production of this endocannabinoid‐like molecule.

**FIGURE 3:**
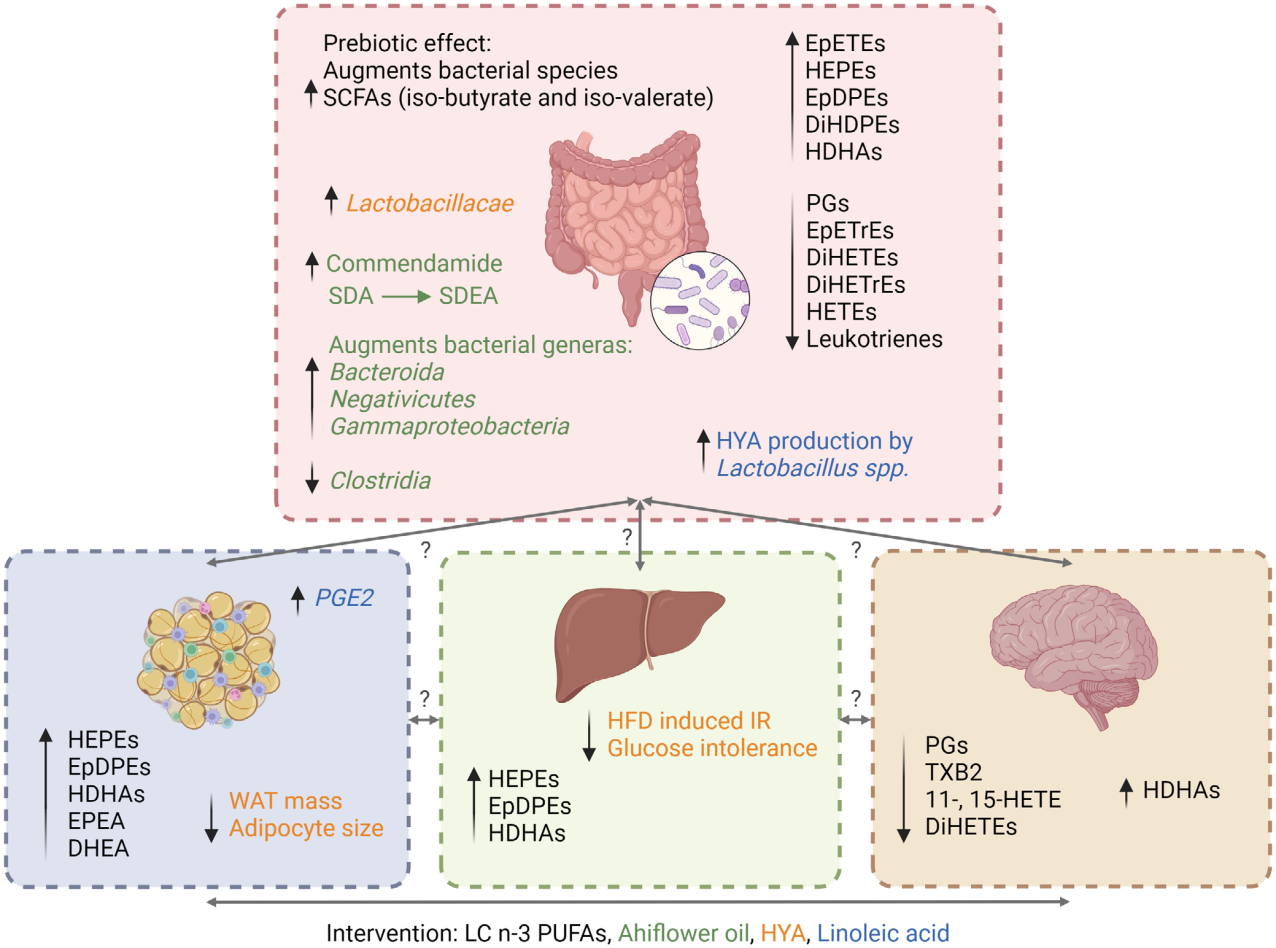
Dietary lipid regulation of intestinal microbiome and oxylipins across the gut‐linked axes. Dietary lipid intervention has been shown to alter gut microbiome and intestinal, adipose, hepatic and brain oxylipin profiles. However, the association between augmented microbiota and oxylipins, and mechanisms of cross‐regulation are not understood, particularly in response to LC n‐3 PUFA. Ahiflower oil intervention highlights an emerging relationship between intestinal microbiota and intestinal endocannabinoid synthesis, and intervention with the linoleic acid microbial metabolite HYA, demonstrates a relationship between oxylipins, intestinal microbiota and metabolic health. In addition, linoleic acid intervention provides further evidence of microbial oxylipin synthesis in response to dietary lipids. Further investigation is required to understand the mechanisms of crosstalk underpinning these emerging relationships and how these may be targeted to improve health. DHEA, docosahexaenoyl ethanolamide; DiHDPEs, dihydroxy‐docosapentaenoic acids; DiHETEs, dihydroxy‐eicosatetraenoic acids; DiHETrEs, dihydroxy‐eicosatrienoic acids; EPEA, eicosapentaenoyl ethanolamide; EpDPEs, epoxy‐docosapentaenoic acids; EpETEs, epoxy‐eicosatetraenoic acid; EpETrEs, epoxy‐eicosatrienoic acids; HDHAs, hydroxy‐docosahexaenoic acids; HEPEs, hydroxy‐eicosapentaenoic acids; HETEs, hydroxy‐eicosatetraenoic acids; HFD, high‐fat diet; HYA, 10‐hydroxy‐cis‐12‐octadecenoic acid; IR, insulin resistance; LC n‐3 PUFAs, long‐chain omega‐3 polyunsaturated fatty acids; SCFAs, short chain fatty acids; PGs, prostaglandins; SDA, stearidonic acid; SDEA, stearidonoyl ethanolamide; TXB2, thromboxane B2; WAT, white adipose tissue. Created in BioRender. Fisk (2024) BioRender.com/h30w417.

This emerging evidence supports cross‐regulation of the gut microbiome and oxylipins spanning gut‐linked axes and cardiac‐splenic axis, along with potential for improvements to inflammation and metabolic health through dietary intervention. Further investigation is required to evaluate translation to humans and further understand the complex relationship between gut microbiota, oxylipin signalling and co‐regulation of several pathophysiological systems. Figure 3 provides a graphical summary depicting the regulation of intestinal microbiome and oxylipins across the gut‐linked axes by dietary lipid intervention.

## FUTURE PERSPECTIVES: HETEROGENEITY IN OXYLIPIN RESPONSES AND THE IMPLICATION OF HOST GENOME

In addition to the variation observed in microbiome composition, other factors contributing to the heterogeneity observed in evidence obtained from clinical studies include age, sex, baseline metabolic status, longevity of obesity and metabolic complication, ethnicity and genetics (Shaikh et al., 2024). These require consideration when devising clinical studies to evaluate the use of nutritional therapeutics, and development of therapeutics with intent to modify microbiota, oxylipin signalling and associated inflammation and metabolic health.

The host genome is emerging as a significant modifier of oxylipin levels and response to dietary lipid intervention. Polymorphisms in CYP450 enzymes which metabolise PUFAs to oxylipins have been identified in individuals living with type 2 diabetes (Rabiee et al., 2018), and variation within the FADS gene locus (FADS genes encoding fatty acid desaturase enzymes) was shown to be associated with PUFA‐containing lipids and signalling molecules including 2‐AG (Chilton et al., 2021). Variation in the frequency of FADS gene cluster variants has been observed across ethnicities with the highest frequency in African, mixed African and South Asian populations, mid‐range frequency in European and East Asian populations, and the lowest frequency in Native American populations (Chilton et al., 2021).

In addition, high population variance has been identified in EPA and resolvin E1 metabolising genes. Individuals carrying a C‐allele in the rs1878022 polymorphism of the ERV1/ChemR23 gene which encodes for the resolvin E1 receptor had lower levels of inflammatory cytokines in adipose tissue (IL‐6) and plasma (IL‐6, IFN‐α, IL‐15, IL‐1ra, IL‐10, GM‐CSF and VEGF) and had enhanced leukocyte responsiveness to resolvin E1 (López‐Vicario et al., 2017). C‐allele carriers also exhibited decreased TAG/HDL ratio which is a surrogate marker of insulin resistance and predictor of fatty liver. This suggests this EVR1/ChemR23 variant is protective against obesity‐associated inflammation (López‐Vicario et al., 2017). In addition to ERV1/ChemR23, data mining of the ESMBL database, including the dbSNP archive, has identified single nucleotide polymorphisms (SNPs) in CYP450 genes (Pal et al., 2020). These genes are involved in the EPA‐resolvin E1 pathway and the identification of SNPs in close proximity on the same chromosome indicates high probability of genetic linkage in many of these variants that can potentially influence metabolism of EPA and derived oxylipins (Pal et al., 2020). This was discussed in support of variation observed in the improvement of insulin resistance in HFD‐fed mice treated with resolvin E1 (300 ng/day) for 4 consecutive days (Pal et al., 2020). Resolvin E1 treatment in C57BL/6J mice, resulted in uniform improvement to fasting glucose and insulin, however, treatment in diet‐induced obese (DIO) mice which mimic human genetic diversity and variability, resulted in improvement to fasting glucose and insulin in only half the animals (Pal et al., 2020). This may be contributed to by associated variation observed in EPA and resolvin E1 metabolising enzymes, which can be identified from data mining and will require identification of SNPs in the future.

In addition, a study utilising genome‐wide analysis in children at risk of developing type‐2 diabetes identified several SNPs to be associated with oxylipin levels (Buckner et al., 2023). The SNP rs143070873 was associated with the LA‐derived oxylipins 9‐HODE and 13‐S‐HODE, respectively, and a locus between MIR1302‐7 and LOC100131146, rs10118380, and an intronic variant in TRPM3 was associated with the AA‐derived oxylipin 11‐HETE (Buckner et al., 2023). The loci interact with PLA2 which is involved in the hydrolysis of fatty acids at the sn‐2 position of phospholipids to release free fatty acids, a crucial step in oxylipin biosynthesis (Buckner et al., 2023). SNPs have also been identified in COX and LOX enzymes involved in the metabolism of PUFAs to oxylipins and associated with poorer metabolic and health outcomes. For example, the rs5275 variant in COX‐2 is associated with type‐2 diabetes in which altered oxylipin concentrations are observed (Ozbayer et al., 2018), and COX‐2 G‐765C polymorphism is associated with increased stroke risk in an African American population (Kohsaka et al., 2008). These associations are similarly seen with SNPs in LOX enzymes which are associated with increased BMI and fat mass (SNPs in ALOX5 (Šerý et al., 2016) and ALOX12), (Xiao et al., 2011) and increased risk of CVD and CAD (SNPs in ALOX5; Šerý et al., 2016) and ALOX15 (Kaur et al., 2018; Zhao et al., 2012). As described earlier in this review, changes in the concentrations of tissue‐specific oxylipins with obesity and cardiac events are observed which may be contributed to by these SNPs. What remains unknown and requires further investigation is whether these genetic variants in LOX and COX enzymes are causative of altered oxylipin profiles in advance of disease development, how altered oxylipins due to these variants contribute to the development and progression of the associated diseases, and if they affect dietary LC n‐3 PUFA metabolism resulting in a lack of associated health benefits from these lipids.

Apolipoprotein E (APOE) genotype has been observed to affect oxylipin response to LC n‐3 PUFA. An intervention study examined the response of plasma oxylipins to EPA and DHA supplementation according to APOE genotype (Saleh et al., 2021) in which healthy adults were given doses of EPA + DHA equating to intakes of 1–4 portions of oily fish per week for 12 months. A significant APOE*LC n‐3 PUFA dose effect was observed in the response of 19‐ and 20‐HEPE and differential responses in oxylipins were identified between APOE alleles (Saleh et al., 2021). Greater increases in HEPEs, HDHAs, DiHETEs and DiHDPEs were observed in APOE4 allele carriers in comparison to APOE3/E3 carriers in response to EPA + DHA supplementation (Saleh et al., 2021). The greatest difference in an EPA‐derived metabolite was 8‐HEPE (1474% in E4 allele carriers vs. 477% in E3) and in a DHA‐derived metabolite was 10‐HDHA (597% in E4 carriers vs. 247% in E3 carriers) (Saleh et al., 2021).

Together, this evidence highlights the need for consideration of host genome as a modifier of oxylipin profile, response of oxylipins to dietary lipid intervention and potential health outcomes associated with modification of oxylipin profile.

Finally, we would like to draw attention to a recent review discussing factors that may impact accurate measurement of oxylipins and interpretation of data (Parchem et al., 2024). Parchem et al. eloquently and comprehensively discuss updates to oxylipin metabolism, their different forms in circulation (esterified and non‐esterified), impact of biological matrices (plasma vs. serum, cells and extracellular vesicles), their bioavailability, impact of analytical parameters (sampling, storage, extraction and analysis) and challenges to interpreting biological significance of oxylipins (Parchem et al., 2024). These areas are of crucial importance in continuing to advance the field of oxylipin research, along with considerations for integration of multi‐platform omics data.

## CONCLUSION

Several aspects of inflammation and immunity require communication between organs and pathophysiological systems which are often linked in disease. As such, augmented microbiome and oxylipin profiles are observed across multiple organs in several disease pathologies including cardiovascular and neurological diseases, and metabolic syndrome‐associated conditions (obesity and MASLD). The aetiology of these diseases is complex and involves multiple mechanisms of communication. The cardiosplenic axis and several gut‐linked axes are emerging systems of crosstalk implicated in disease, and communication via oxylipin and gut microbiota is an emerging mechanism by which this crosstalk is established and maintained. This highlights an accessible route for therapeutic intervention for the treatment and prevention of disease through nutrition. Dietary intervention with lipids and or probiotic organisms has the potential to modify the gut microbiome and oxylipin profiles to improve health outcomes relevant to the diseases mentioned above. However, key mechanisms by which the gut microbiome and oxylipins are co‐regulated and communicate remain unknown. Further investigation is required to advance our understanding of this relationship and impact on health outcomes associated with modification of these systems. Human intervention studies investigating the use of LC n‐3 PUFAs and/or probiotic organisms are needed to translate evidence from animal studies and to identify and understand key mechanisms by which a relationship between these two systems is established. Furthermore, human studies are required to further understand the biological and clinical relevance of this crosstalk and co‐regulation by nutritional intervention.

## AUTHOR CONTRIBUTIONS

H.L.F.: conceptualisation, original manuscript drafting, figure preparation and manuscript revision; S.R.S.: conceptualisation and manuscript revision.

## CONFLICT OF INTEREST STATEMENT

H.L.F. declares no competing interests. S.R.S. has received industry support (Metagenics, Wiley Companies) for research and organisation of conferences related to n‐3 and n‐7 fatty acids.

## Data Availability

Data sharing not applicable to this article as no datasets were generated or analysed during the current study.
